# Camera Calibration Optimization Algorithm Based on Nutcracker Optimization Algorithm

**DOI:** 10.3390/s25113521

**Published:** 2025-06-03

**Authors:** Lei Li, Zelong Xiao, Taiyang Hu

**Affiliations:** School of Electronic and Optical Engineering, Nanjing University of Science and Technology, Nanjing 210094, China; 220104011104@njust.edu.cn (L.L.); tyhu@njust.edu.cn (T.H.)

**Keywords:** camera calibration, Zhang’s calibration method, starling optimization algorithm, chaotic mapping, sine cosine optimization

## Abstract

Camera calibration is a core task in computer vision. Traditional calibration methods usually achieve approximate solutions due to the complexity of solving nonlinear equations, resulting in reprojection errors. This article proposes a camera calibration optimization algorithm based on the Starling-Inspired Strategy optimization algorithm, which improves calibration accuracy and stability by combining chaotic mapping and sine cosine optimization strategies. First, we constructed a real camera calibration image dataset that includes various calibration scenarios and calculated the initial values of camera calibration parameters based on Zhang’s calibration method. Then, we established a reprojection error model to evaluate the accuracy of the calibration parameters. Finally, we reduced the reprojection error through a hybrid optimization method based on the Steller Jay optimization algorithm. Experimental results show that our algorithm significantly reduces the reprojection error and improves the camera calibration accuracy.

## 1. Introduction

Camera calibration is a fundamental task in computer vision, crucial for associating 2D image coordinates with the 3D world coordinate system. It determines a camera’s internal parameters (focal length, principal point, and distortion coefficients) and external parameters (position and orientation), which are vital for applications like 3D reconstruction, object recognition, augmented reality (AR), and autonomous driving. However, traditional calibration methods often suffer from local optima in solving nonlinear problems, leading to significant reprojection errors and compromised visual task performance [1,2,3].

The Tsai camera model and its two-step calibration method hold milestone significance in computer vision by reducing iterations and achieving faster computation through linear parameter solving. However, its simplistic distortion model limits effectiveness, especially with complex image distortion. To address this, researchers have developed advanced distortion models, such as higher-order polynomial fitting and machine learning-based methods, to enhance the accuracy of correction [4,5,6].

Self-calibration, especially as implemented in photogrammetric bundle adjustment, is a well-established and widely adopted technique. It achieves sub-pixel accuracy (e.g., ∼0.025 px) when the imaging network satisfies proper geometric conditions (e.g., convergent views and roll angle diversity) [7,8]. The focus of our work is not to replace such rigorous models but to explore optimization schemes that may assist parameter refinement or be deployed when traditional methods are limited by data or computation.

In recent years, many optimization-based calibration approaches have been proposed to improve the robustness and precision of intrinsic and extrinsic parameter estimation. Classical non-linear optimization methods such as Levenberg–Marquardt remain dominant [8], but evolutionary algorithms, including Particle Swarm Optimization (PSO) [9], Differential Evolution (DE) [10], Genetic Algorithms (GA) [11], and more recent metaheuristics like the Sparrow Search Algorithm (SSA) [12] and Sine Cosine Algorithm (SCA) [13], have been produced to reduce reprojection errors and enhance global search capabilities. Furthermore, several deep learning-based calibration frameworks [6,14] have emerged, which are capable of regressing camera parameters directly from image data, though they often require extensive data and lack interpretability.

Researchers actively explore the application of various optimization algorithms in camera calibration to improve calibration accuracy and stability. For example, Du Shuai et al. combined the adaptive differential evolution algorithm with the seagull algorithm, based on the seagull algorithm, to optimize multi-camera calibration. This algorithm can achieve good results on multi-parameter problems and effectively avoid falling into local optima [15]. To improve parameter calibration’s low accuracy and poor re-usability, Guan Chunxi et al. proposed a camera parameter calibration optimization method based on the chaotic sparrow search algorithm (SSA) [16]. Fu Wei et al. introduced an adaptive judgment factor to control the improved algorithm of differential evolution (IDE) and particle swarm optimization (PSO) algorithm in each iteration process, solving the problem of multiple parameters in traditional camera calibration algorithms. The proposed algorithm can ensure the diversity and effectiveness of the population’s individual evolution [17]. Sha Xinyu et al. proposed an enhanced hybrid optimization method using the principles of differential evolution and particle swarm optimization and then applied it to camera calibration. The proposed algorithm improves the accuracy and convergence speed of camera calibration. It effectively solves the problem of other algorithms falling into local optimal values due to image distortion [18]. These studies demonstrate the optimization algorithms’ wide application and potentiality in camera calibration, providing new directions for future research.

In this paper, the Nutcracker Optimization Algorithm (NOA) is introduced into the field of camera calibration, and a new Hybrid Optimization Algorithm (NCS) is proposed to optimize the internal parameters and distortion coefficients of the camera by combining chaos mapping and sine and cosine optimization strategies. By simulating the foraging, storage, and retrieval behaviors of Star Crows, the algorithm realizes the global search and local optimization of the camera calibration parameters and improves the accuracy and stability of the calibration. To overcome the defect that traditional optimization algorithms may converge to local optima, this paper introduces Chebyshev chaos mapping and sine and cosine optimization strategies based on the Star Crow optimization algorithm. Chaos mapping enhances the randomness and diversity of the algorithm, while the sine and cosine optimization strategy improves the local development ability of the algorithm; the combination of the two effectively improves the global search ability and robustness of the algorithm. In this paper, experiments verify the effectiveness of the proposed camera calibration optimization algorithm based on the Star Crow optimization algorithm. Experimental results show that, compared with the slime mold algorithm (SMA), the slime optimization algorithm (ORSMA), and the simple Star Crow optimization algorithm (NOA), the improved NCS algorithm has an excellent performance in reducing the reprojection error, has a higher convergence speed and better global search ability, and provides a new and effective solution for camera calibration optimization.

## 2. NCS Algorithm Principle

### 2.1. NOA Algorithm Principle

In 2023, M. Abdel Basset and his colleagues were inspired by the natural behavior of Clark’s nutcrackers and developed a new algorithm called the Nutcracker Optimization Algorithm (NOA) [19]. The NOA algorithm simulates two main behavioral strategies of the nutcrackers: (i) foraging and storing strategy; (ii) cache searching and recovery strategy. Assuming the population size is *N* and the problem dimension is *D*, the initialization formula is

(1) $$\vec{X_{i,j}^{t}}=\left(\vec{U_{j}}-\vec{L_{j}}\right)\cdot \vec{RM}+\vec{L_{j}}$$

where $X_{i,j}$ is the *j*-dimensional variable representing individual *i*, i=1,2,…,N; j=1,2,…,D, *t* is the current number of iterations, $\vec{U_{j}}$ and $\vec{L_{j}}$ denote the next and previous *j*-dimensional variables, respectively. $\vec{RM}$ is a random vector in the interval [0,1]. During the foraging phase, the Star Crow hunts for food in the search space:

(2) $$\vec{X_{i}^{t+1}}=\left\{\begin{array}{cc} X_{i,j}^{t} & \mathrm{if}\;\tau_{1}<\tau_{2},\\ \left\{\begin{array}{cc} X_{m,j}^{t}+\gamma \cdot \left(X_{A,j}^{t}-X_{B,j}^{t}\right)\\ \;+\mu \cdot (r^{2}\cdot U_{j}-L_{j}) & \mathrm{if}\;t\leq T_{max}/2.0,\\ X_{C,j}^{t}+\mu \cdot \left(X_{A,j}^{t}-X_{B,j}^{t}\right)\\ \;+\mu \cdot \left(r_{1}<\delta \right)\cdot \left(r^{2}\cdot U_{j}-L_{j}\right) & \mathrm{otherwise} \end{array}\right. & \mathrm{otherwise} \end{array}\right.$$

where $X_{i}^{t+1}$ is the new position of the *i*-th nutcracker at iteration t+1, $X_{m,j}^{t}$ is the *j*-th dimension mean of all solutions in the current population at iteration *t*, and A,B,C are three different Star Crow individuals randomly selected from the population, respectively. $T_{max}$ is the maximum number of iterations. The parameter γ is a random number generated from the Levy flight function. δ is set to 0.05. $\tau_{1},\tau_{2},\tau_{3},r,r_{1}$ are random numbers between [0,1], $\tau_{4}$ is a random number that obeys a normal distribution, $\tau_{5}$ is a random number generated by Levy flight, and μ is the number generated based on $\tau_{3}$, $\tau_{4}$ and $\tau_{5}$ as Equation (3), and the initial reference point can be generated by Equation (1).

(3) $$\mu =\left\{\begin{array}{cc} \tau_{3} & \mathrm{if}\;r_{1}<r_{2}\\ \tau_{4} & \mathrm{if}\;r_{2}<r_{3}\\ \tau_{5} & \mathrm{if}\;r_{1}<r_{3} \end{array}\right.$$

During the storage phase, the crow first transports the food obtained in the previous stage to a temporary storage location:

(4) $$\vec{X_{i}^{t+1(new)}}=\left\{\begin{array}{cc} \vec{X_{i}^{t}}+\mu \cdot \left(\vec{X_{best}^{t}}-\vec{X_{i}^{t}}\right)\cdot \left|\lambda \right|\\ \;+r_{1}\cdot \left(\vec{X_{A}^{t}}-\vec{X_{B}^{t}}\right), & \mathrm{if}\;\tau_{1}<\tau_{2},\\ \vec{X_{best}^{t}}+\mu \cdot \left(\vec{X_{A}^{t}}-\vec{X_{B}^{t}}\right) & \mathrm{if}\;\tau_{1}<\tau_{3},\\ \vec{X_{best}^{t}}\cdot l & \mathrm{otherwise} \end{array}\right.$$

where $X_{i}^{t}$ is the current position of the *i*-th nutcracker in the current iteration *t*, $X_{best}^{t}$ is the optimal individual of the current population, and λ is the random number generated by the Levy flight, *l* is the factor that decreases linearly from 1 to 0 in the development behavior of NOA. The conversion between foraging and storage is performed according to Equation (5) to maintain the balance between caching and recovery.

(5) $$\vec{X_{i}^{t+1}}=\left\{\begin{array}{cc} \mathrm{Equation}\;(2) & \mathrm{if}\;\varphi >P_{a_{1}},\\ \mathrm{Equation}\;(4) & \mathrm{otherwise} \end{array}\right.$$

where φ is a random number between [0,1], and $P_{a_{1}}$ is a probability value, decreasing from 1 to 0. Only one of the two strategies is executed. The cache search and recovery strategy is based on two reference points (RPs) of individual crows. In short, there are two reference points that the Star Crow chooses to remember where the food is stored. The two reference points are calculated as

(6) $${\vec{RP}}_{i,1}^{t}=\left\{\begin{array}{cc} {\vec{X}}_{i}^{t}+\alpha \cdot \cos\left(\theta \right)\cdot \left({\vec{X}}_{A}^{t}-{\vec{X}}_{B}^{t}\right)+\alpha \cdot RP & \mathrm{if}\;\theta =\pi /2\\ {\vec{X}}_{i}^{t}+\alpha \cdot \cos\left(\theta \right)\cdot \left({\vec{X}}_{A}^{t}-{\vec{X}}_{B}^{t}\right) & \mathrm{otherwise} \end{array}\right.$$

(7) $$\vec{RP_{i,2}^{t}}=\left\{\begin{array}{cc} \vec{X_{i}^{t}}+(\alpha \cdot \cos\left(\theta \right)\cdot \left(\left(\vec{U}-\vec{L}\right)\cdot \tau_{3}+\vec{L}\right)\\ \;+\alpha \cdot RP)\cdot {\vec{U}}_{2} & \mathrm{if}\;\theta =\pi /2\\ \vec{X_{i}^{t}}+\alpha \cdot \cos\left(\theta \right)\cdot \left(\left(\vec{U}-\vec{L}\right)\cdot \tau_{3}+\vec{L}\right)\cdot {\vec{U}}_{2} & \mathrm{otherwise} \end{array}\right.$$

(8) $${\vec{U}}_{2}=\left\{\begin{array}{cc} 1 & {\vec{r}}_{2}<P_{rp}\\ 0 & \mathrm{otherwise} \end{array}\right.$$

(9) $$\alpha =\left\{\begin{array}{cc} {\left(1-\frac{t}{T_{max}}\right)}^{\frac{2t}{T_{max}}} & \mathrm{if}\;r_{1}>r_{2}\\ {\left(\frac{t}{T_{max}}\right)}^{\frac{t}{2}} & \mathrm{otherwise} \end{array}\right.$$

where α ensures the regular convergence of the NOA, allowing Star Crow to improve its RP selection in the next generation, θ is the random radian between [0,π], ${\vec{r}}_{2}$ is the maximum vector between [0,1], and $P_{rp}$ determines the probability of globally exploring other regions within the search space.

During the cache search phase,

(10) $$X_{i,j}^{t+1}=\left\{\begin{array}{cc} X_{i,j}^{t} & \mathrm{if}\;\tau_{3}<\tau_{4},\\ X_{i,j}^{t}+r_{1}\cdot \left(X_{best,j}^{t}-X_{i,j}^{t}\right)+r_{2}\cdot \left(\vec{RP_{i,1}^{t}}-X_{C,j}^{t}\right) & \mathrm{otherwise} \end{array}\right.$$

(11) $$X_{i,j}^{t+1}=\left\{\begin{array}{cc} X_{i,j}^{t} & \mathrm{if}\;\tau_{5}<\tau_{6},\\ X_{i,j}^{t}+r_{1}\cdot \left(X_{best,j}^{t}-X_{i,j}^{t}\right)+r_{2}\cdot \left(\vec{RP_{i,2}^{t}}-X_{C,j}^{t}\right) & \mathrm{otherwise} \end{array}\right.$$

(12) $${\vec{X}}_{i}^{t+1}=\left\{\begin{array}{cc} \mathrm{Equation}\;(10) & \mathrm{if}\;\tau_{7}<\tau_{8},\\ \mathrm{Equation}\;(11) & \mathrm{otherwise} \end{array}\right.$$

where $\tau_{i}\left(i=6,7,8\right)$ are all random numbers between [0,1]. During the recovery phase:

(13) $${\vec{X}}_{i}^{t+1}=\left\{\begin{array}{cc} {\vec{X}}_{i}^{t} & \mathrm{if}\;f\left(\vec{X_{i}^{t}}\right)<f\left(\vec{RP_{i,1}^{t}}\right)\\ \vec{RP_{i,1}^{t}} & \mathrm{otherwise} \end{array}\right.$$

(14) $${\vec{X}}_{i}^{t+1}=\left\{\begin{array}{cc} {\vec{X}}_{i}^{t} & \mathrm{if}\;f\left(\vec{X_{i}^{t}}\right)<f\left(\vec{RP_{i,2}^{t}}\right)\\ \vec{RP_{i,2}^{t}} & \mathrm{otherwise} \end{array}\right.$$

(15) $${\vec{X}}_{i}^{t+1}=\left\{\begin{array}{cc} \mathrm{Equation}\;(13) & \mathrm{if}\;f\left(\vec{RP_{i,1}^{t}}\right)<f\left(\vec{RP_{i,2}^{t}}\right)\\ \mathrm{Equation}\;(14) & \mathrm{otherwise} \end{array}\right.$$

### 2.2. Basic Principles of Chaos Mapping Algorithms

Among various chaotic systems (e.g., Logistic, Tent, Gauss, and Chebyshev), the Chebyshev map was selected due to its favorable properties for population-based optimization. It exhibits high sensitivity to initial conditions, strong ergodicity, and a higher Lyapunov exponent than most other chaotic systems, enabling the generation of diverse initial populations. Compared with the logistic map, the Chebyshev map avoids short periodic orbits and better maintains randomness, which is critical for escaping local optima during optimization. The chaotic map adopted in this study is the Chebyshev map, which is based on Chebyshev polynomials and exhibits strong randomness and nonlinearity. Its formulation is given as follows:

(16) $$\begin{array}{cc} \; & x\left(i+1\right)=\cos(a\cdot \cos^{-1}\left(x\left(i\right)\right))\\ \; & P_{a_{2}}=0.4x\left(i+1\right) \end{array}$$

where *a* is a constant (commonly taken) set to 4, and $P_{a_{2}}$ is a probability value. The range of chaotic orbit state values is (−1,1), which ensures that the generated chaotic sequence remains bounded and can be effectively scaled for initializing population values within defined limits of the optimization variables.

### 2.3. Basic Principles of the Sine and Cosine Optimization Strategy

While NOA performs well in global exploration through foraging and caching behavior, its local exploitation ability may weaken in the later stages of iteration. The integration of the SCA addresses this issue by introducing a deterministic yet oscillatory update mechanism that fine-tunes candidate solutions near promising regions. This hybrid strategy leverages the global exploration ability of NOA and the local refinement of SCA, thereby enhancing the overall balance between exploration and exploitation [20].

The Sine Cosine Optimization Algorithm (SCO) is a stochastic optimization algorithm characterized by high flexibility, simple principles, ease of implementation, and conveniently applied to optimization problems in various fields [20]. Its specific update equation is the following:

(17) $$X_{i}^{t+1}=\left\{\begin{array}{cc} X_{i}^{t}+r_{1}\cdot \sin\left(r_{2}\right)\cdot \left|r_{3}P_{i}^{t}-X_{i}^{t}\right| & \mathrm{if}\;r_{4}<0.5\\ X_{i}^{t}+r_{1}\cdot \cos\left(r_{2}\right)\cdot \left|r_{3}P_{i}^{t}-X_{i}^{t}\right| & \mathrm{if}\;r_{4}>0.5 \end{array}\right.$$

where

(18) $$r_{1}=a-t\frac{a}{T_{max}},$$

$r_{2}$ is a random number from 0 to 2π, $r_{3}$ is a random number between 0 and 2, $r_{4}$ is a random number from 0 to 1, $P_{i}^{t}$ denotes the position of the *i*-dimension of the optimal individual position variable at *t* iterations.

## 3. Design and Application of Camera Internal Parameter Optimization Based on NOA

**Figure 1:**
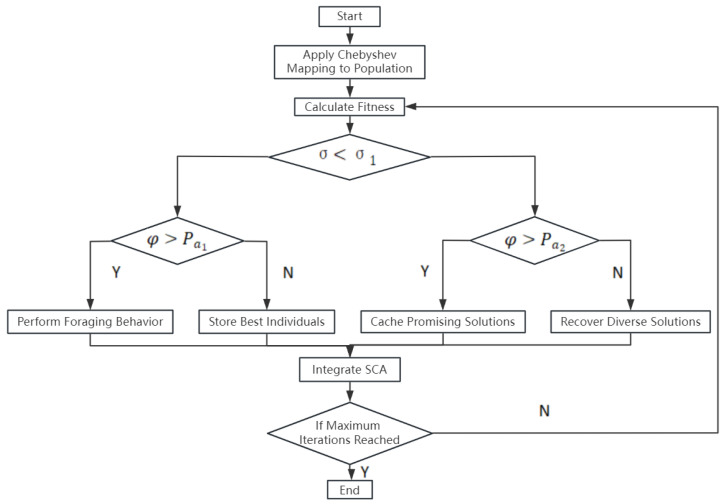
Algorithm flow of parameter optimization.

In the design process, the Star Crow optimization algorithm cleverly combines chaos mapping and sine and cosine optimization algorithms to improve its search efficiency and global optimization ability. The flow chart of the internal control optimization algorithm is shown in Figure 1.

### 3.1. Camera Imaging Model and Parameter Definition

Camera calibration aims to determine the precise mapping between three-dimensional (3D) world coordinates and two-dimensional (2D) image coordinates. The widely used pinhole camera model is typically adopted to describe this imaging process, consisting of three components: intrinsic parameters, extrinsic parameters, and distortion parameters [21].

#### 3.1.1. Intrinsic Parameters

The intrinsic parameters define the internal characteristics of the camera, which relate the camera coordinate system to the image coordinate system. The intrinsic parameter matrix *K* is given by the following:

(19) $$K=\left[\begin{array}{ccc} f_{x} & 0 & u_{0}\\ 0 & f_{y} & v_{0}\\ 0 & 0 & 1 \end{array}\right]$$

where $f_{x}$ and $f_{y}$ represent the focal lengths in pixels along the *x*-axis and *y*-axis, respectively, and $\left(u_{0},v_{0}\right)$ denote the coordinates of the principal point, ideally located near the center of the image sensor. In an ideal case with square pixels, $f_{x}$ and $f_{y}$ are approximately equal. The principal point $\left(u_{0},v_{0}\right)$ is typically near half of the image width and height.

#### 3.1.2. Extrinsic Parameters

The extrinsic parameters describe the rigid body transformation from the 3D world coordinate system to the 3D camera coordinate system, comprising a rotation matrix R and a translation vector T. The projection relationship can be written as follows:

(20) $$s\left[\begin{array}{c} x\\ y\\ 1 \end{array}\right]=K\left[\begin{array}{cc} R & T \end{array}\right]\left[\begin{array}{c} X\\ Y\\ Z\\ 1 \end{array}\right]$$

where (X,Y,Z) are the world coordinates, (x,y) are the normalized image coordinates, and *s* is a scale factor.

#### 3.1.3. Distortion Parameters

In practical imaging, optical systems inevitably introduce distortion. Two main types of distortion are modeled: Radial distortion, causing straight lines to appear curved, typically modeled by coefficients $k_{1}$, $k_{2}$, and $k_{3}$; Tangential distortion, resulting from imperfect alignment of the lens and image plane, modeled by coefficients $p_{1}$ and $p_{2}$. The distorted image coordinates $\left(x_{d},y_{d}\right)$ can be expressed as follows:

(21) $$\left\{\begin{array}{cc} r^{2} & =x^{2}+y^{2}\\ x_{d} & =x\left(1+k_{1}r^{2}+k_{2}r^{4}+k_{3}r^{6}\right)+2p_{1}xy+p_{2}\left(r^{2}+2x^{2}\right)\\ y_{d} & =y\left(1+k_{1}r^{2}+k_{2}r^{4}+k_{3}r^{6}\right)+p_{1}\left(r^{2}+2y^{2}\right)+2p_{2}xy \end{array}\right.$$

where *r* is the radial distance from the principal point. The accurate modeling and compensation of these distortions are essential for achieving high-precision camera calibration.

#### 3.1.4. Distortion Parameters

The objective of camera calibration is to minimize the reprojection error, defined as the Euclidean distance between the observed image points and the projected points. The cost function can be expressed as follows:

(22) $$\min_{K,D,R,T}\Sigma_{i=1}^{N}{∥P_{i}^{obs}-P_{i}^{proj}∥}^{2}$$

where $P_{i}^{obs}$ is the observed image point, $P_{i}^{proj}$ is the projected point computed from the estimated parameters, *N* is the total number of feature points, and *D* represents the distortion parameters. In this study, the widely recognized camera calibration method proposed by Zhang [21] is adopted, and practical implementation is carried out using the OpenCV library [22].

### 3.2. The Objective Function Is Established

The objective function of the camera calibration problem is established as follows:

(23) $$f=\Sigma_{i=1}^{N}∥p_{ij}-p\left(f_{x},f_{y},u_{0},v_{0},k_{1},k_{2},k_{3},p_{1},p_{2},R,T\right)∥$$

$p_{ij}$ is the actual pixel coordinate of the *j*-th corner; *p* is the calculated pixel coordinates;$f_{x}$, $f_{y}$, $u_{0},v_{0}$ are internal parameters for the camera; $k_{1},k_{2},k_{3},p_{1},p_{2}$ are radial distortion and tangential distortion coefficients; *R* and *T* are image rotation translation matrix, respectively.

### 3.3. Hybrid Algorithm Application

In order to enhance the performance of the original algorithm in terms of exploration and exploitation, a hybrid strategy is adopted in this study. Firstly, chaos mapping is introduced to increase algorithmic randomness and population diversity. By integrating chaotic sequences into the initialization and update phases, the algorithm is enabled to explore the search space more thoroughly and avoid premature convergence. Secondly, the sine and cosine optimization algorithm is embedded into the NOA framework. Leveraging the periodicity and oscillatory nature of the sine and cosine functions, the algorithm can dynamically balance the depth and breadth of the search, thereby achieving a better trade-off between local and global optima.

Firstly, chaos mapping is introduced to enhance algorithm randomness and diversity, and by introducing chaos sequences in the initialization and update process, the algorithm can be explored more extensively and in detail in the search space. Secondly, the sine and cosine optimization algorithm is integrated into the search strategy of NOA, and the periodicity and fluctuation of the sine and cosine function are used to guide the algorithm to balance the depth and breadth of the search process so as to find the best compromise between the local optimal solution and the global optimal solution. This combination not only enhances the adaptability and robustness of NOA but also significantly improves its performance in complex optimization problems. The pseudo-code of the hybrid algorithm is shown in Algorithm 1:
**Algorithm 1** Algorithm NCS Hybrid1:Input parameter: pop, MaxIter, dim2:Output parameter: Best_score3:Function NCS4:Initialize Positions of Individuals in Population         $x\left(i+1\right)=\cos(a\cdot \cos^{-1}\left(x\left(i\right)\right))$5:Calculate *f*6:**if** $\sigma <\sigma_{a_{2}}$ **then**7:   **if** $\varphi <P_{a_{1}}$ **then**8:     Perform Foraging Behavior(individual)9:   **else**10:     Store Best Individuals from Population11:   **end if**12:**else**13:   **if** $\varphi >P_{a_{2}}$ **then**14:     Cache Promising Solutions15:   **else**16:     Recover Diverse Solutions17:   **end if**18:**end if**19:Integrate SCA into Genetic Algorithm20:Update *f*21:**if** Maximum Iterations Reached **then**22:    Break23:**end if**24:**return** Best Solution from Population

## 4. Experiment

### 4.1. Protocol Design

**Figure 2:**
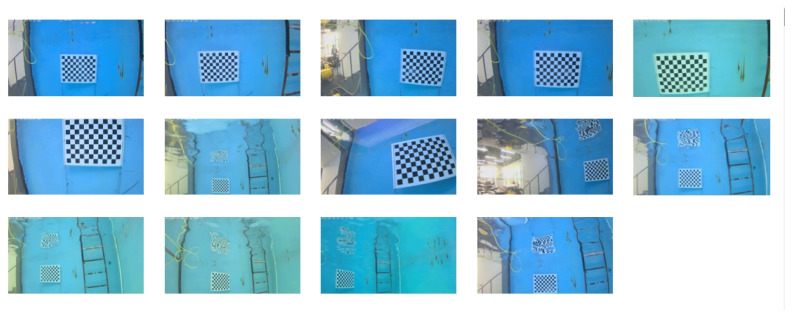
Calibrate the pictures.

All experiments were conducted on a desktop computer equipped with an Intel Core i7-12700F CPU @ 2.10 GHz (Intel Corporation, Wuhan, China), 32 GB RAM, and running Windows 11 with Python 3.10. The optimization algorithms were implemented using the NumPy 1.24.3 and SciPy 1.10.1 libraries. All calibration tasks were carried out using the real images obtained by the Boya Gongdao R1-10Li underwater robot camera. The adopted 1/2.8-inch CMOS camera has a resolution of 1920 × 1080. The NCS algorithm was tested with the following default parameters: population size N=30, maximum iterations T=500, chaotic map parameter a=4, and SCA control parameters α∈[0,2π],β∈[0,1]. These parameters were selected based on preliminary tuning to balance convergence quality and computational cost. The 14 images taken in this experiment are shown in Figure 2, and the calibration of the camera’s internal parameters and distortion coefficients is realized based on these pictures.

### 4.2. Analysis of Experimental Results

**Figure 3:**
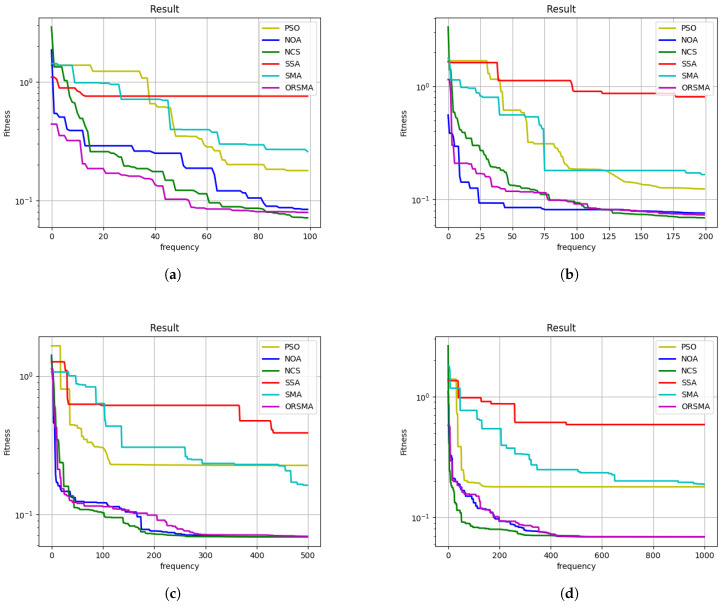
Objective Function Curve: (**a**) 100 iterations. (**b**) 500 iterations. (**c**) 100 iterations. (**d**) 1000 iterations.

To evaluate the optimization performance of different algorithms for camera intrinsic parameter calibration, convergence curves and parameter tables at 100, 200, 500, and 1000 iterations were analyzed. As shown in Figure 3, the Neighborhood-based Chaotic Sine Cosine algorithm (NCS) consistently demonstrates superior convergence behavior compared to other algorithms, including SSA, NOA, PSO, SMA, and ORSMA.

From the convergence profiles, NCS achieves rapid error reduction within the early 50 iterations and continues stable optimization toward lower fitness values, outperforming others in both convergence speed and final accuracy. Table 1 shows the calibration results of Zhang’s calibration method. As shown in Table 2, Table 3, Table 4 and Table 5, at 100 iterations, the calibration error of NCS drops to 0.0714, significantly lower than SSA (0.7586), NOA (0.0841), PSO (0.1787), SMA (0.2584), and ORSMA (0.0793). This trend persists across higher iteration counts, where NCS maintains minimal final errors of 0.0690 at 200 iterations, 0.0687 at 500 iterations, and 0.0687 at 1000 iterations. These results validate that NCS not only accelerates convergence but also achieves higher calibration precision.

Parameter stability across iterations further corroborates NCS’s effectiveness. For instance, the estimated focal lengths $\left(f_{x},f_{y}\right)$ and principal point coordinates $\left(u_{0},v_{0}\right)$ converge consistently with minor fluctuations for NCS, whereas other methods exhibit larger variations or slower stabilization. Additionally, NCS achieves more reasonable distortion coefficients $\left(k_{1},k_{2},p_{1},p_{2},k_{3}\right)$, with reduced overfitting risk and better preservation of the expected radial distortion characteristics.

It is also noteworthy that while ORSMA presents comparable final errors to NCS, its convergence speed is slower, and parameter stability is inferior, especially at early iterations. In contrast, NCS achieves an excellent balance between fast convergence, minimal final error, and stable parameter estimation, making it particularly suitable for camera calibration tasks requiring high precision and efficiency.

Although a strict mathematical proof of convergence is challenging due to the stochastic nature of metaheuristic algorithms, we provide a qualitative convergence analysis. The proposed NCS algorithm combines three modules: chaotic initialization, NOA exploration, and SCA-based refinement. Each module satisfies the necessary conditions for global search completeness.

Regarding computational complexity, let *N* be the population size, *D* the problem dimension, and *T* the number of iterations. Then the overall complexity is O(N·D·T), consistent with standard population-based optimizers. The inclusion of Chebyshev mapping and SCA modules introduces only a small constant overhead per iteration, which is negligible compared to the gains in convergence stability and accuracy.

**Figure 4:**
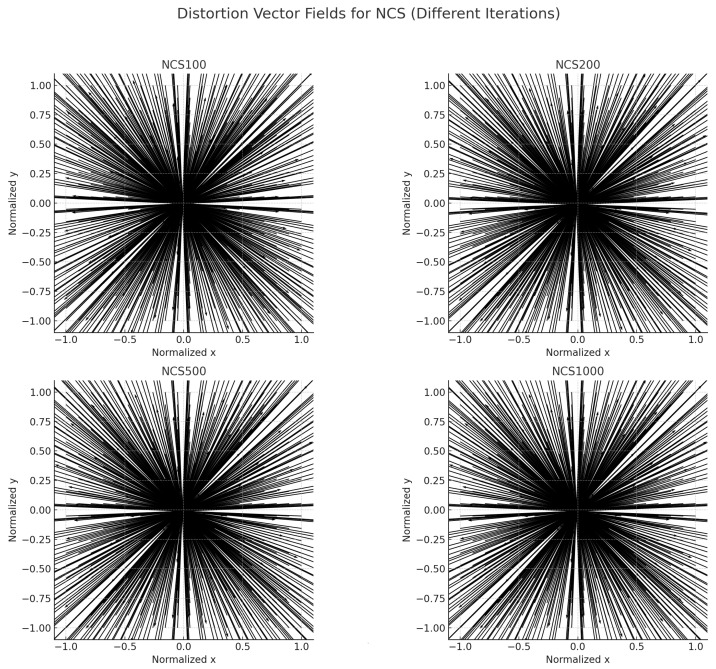
Comparison graph of distortion vector fields with different iteration numbers of NCS.

Figure 4 illustrates the distortion vector fields of the NCS optimization algorithm at different iteration stages (100, 200, 500, and 1000 iterations). Each vector field depicts the direction and magnitude of pixel displacements caused by lens distortion, normalized within the range [−1,1]. Overall, the distortion vectors exhibit a consistent outward radial pattern, indicating a typical barrel distortion characteristic.

As the number of iterations increases, notable improvements in the uniformity and symmetry of the vector fields are observed. Specifically, at 100 iterations (NCS100), the distortion vectors display slight irregularities near the image periphery, and local inconsistencies in vector orientation can be seen. By 200 iterations (NCS200), the distortion vectors become more radially aligned, and local nonuniformities are significantly reduced. At 500 iterations (NCS500), the distortion vectors demonstrate highly symmetrical radial distribution, and the magnitudes of distortion are more smoothly varying with the radius. Finally, at 1000 iterations (NCS1000), the vector field achieves an almost ideal radial expansion pattern, indicating that the distortion correction model has effectively converged. The vector density and orientation become extremely regular, reflecting minimal local deviations and highly uniform distortion compensation.

These results quantitatively and visually validate that increasing the optimization iterations leads to a more consistent and symmetrical distortion model. This not only reduces the overall distortion magnitude but also ensures uniform distortion behavior across the entire image plane, which is critical for high-precision imaging applications.

**Figure 5:**
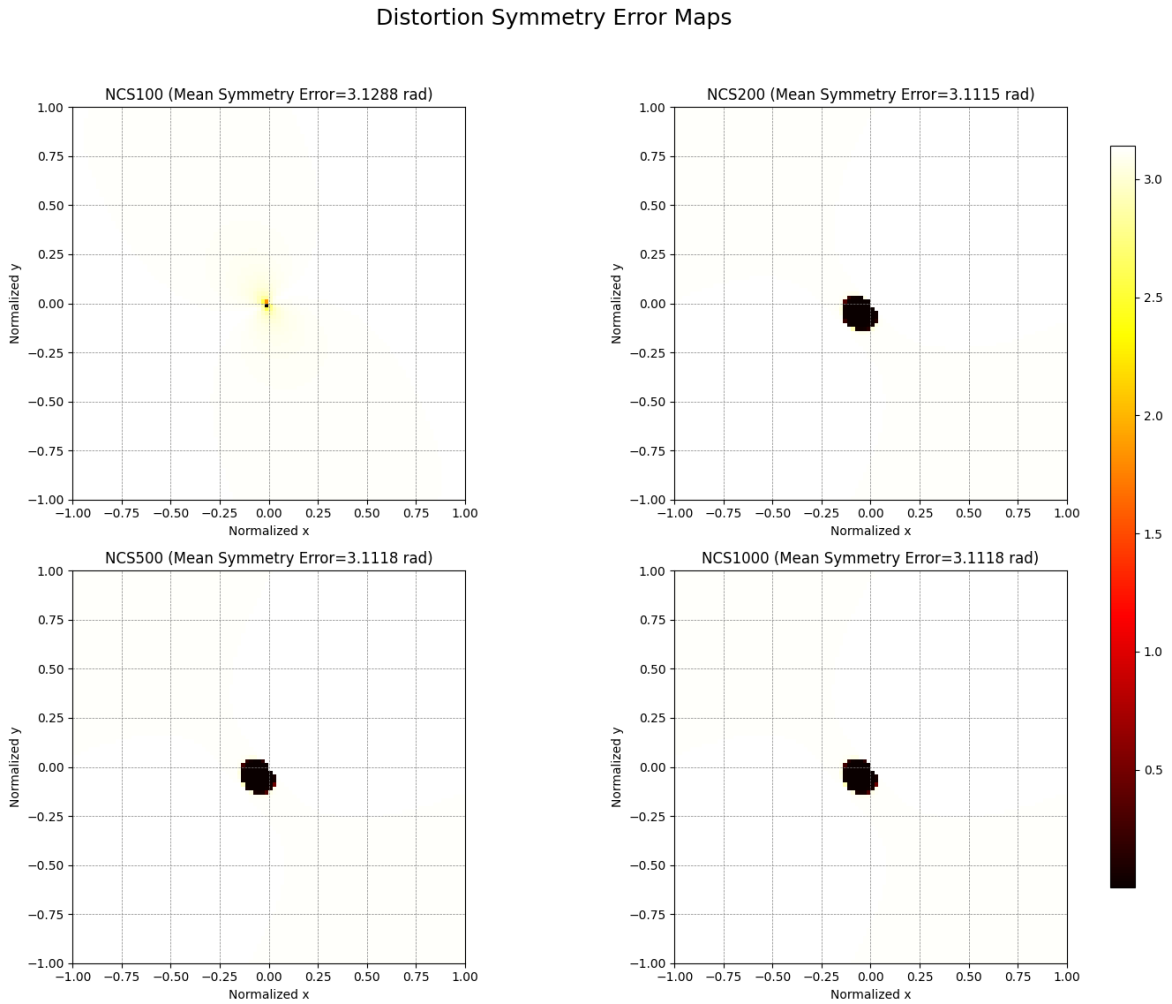
NCS Symmetry error heat maps of each iteration number.

Figure 5 presents the distortion symmetry error maps corresponding to NCS optimization at different iteration stages (100, 200, 500, and 1000 iterations). The color intensity represents the angular deviation (in radians) between the actual distortion direction and the ideal radial direction, serving as a quantitative indicator of distortion field symmetry. Overall, the mean symmetry error remains relatively low across all iterations, indicating that the distortion vectors are closely aligned with the ideal radial direction. From NCS100 to NCS1000, the distortion symmetry error maps reveal a localized concentration of minor angular deviations around the image center, while the majority of the image field maintains excellent radial alignment.

Specifically, at 100 iterations (NCS100), the symmetry error is slightly more dispersed around the center, whereas with increased iterations (NCS200, NCS500, and NCS1000), the errors become increasingly confined and minimal. This trend suggests that as optimization progresses, the distortion field not only reduces its overall magnitude but also improves its angular consistency.

The observed minimal and localized symmetry errors validate the effectiveness of the NCS algorithm in enforcing radial distortion uniformity, which is crucial for maintaining geometric fidelity in high-precision vision applications.

**Figure 6:**
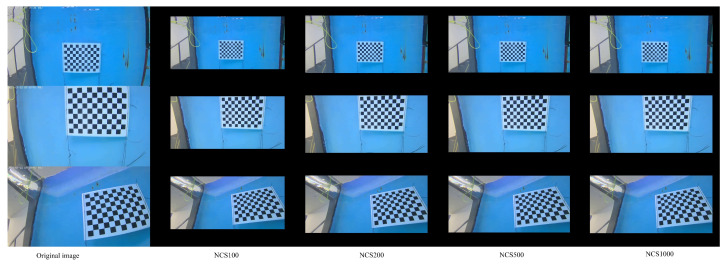
The actual image correction effect diagram under different iteration times of NCS.

In order to assess the influence of iteration numbers on distortion correction performance, a series of underwater checkerboard images were utilized. As shown in Figure 6, each sequence comprised the original distorted image alongside corrected results obtained by applying the NCS optimization algorithm with 100, 200, 500, and 1000 iterations, respectively. It is evident that the application of distortion correction significantly mitigates barrel and pincushion effects. Following 100 iterations, primary distortion removal is achieved; however, minor residual deformations persist, particularly at the image periphery. As the iteration number increases to 200 and 500, the corrected images exhibit improved linearity of the checkerboard edges, with distortions near the boundaries substantially reduced. At 1000 iterations, the distortion is effectively eliminated, yielding a highly regular checkerboard structure. Furthermore, the uniformity of distortion correction across both the central and peripheral regions is enhanced with higher iteration counts, resulting in minimal residual geometric deformation. The observed consistency across multiple viewpoints further verifies the robustness and stability of the NCS algorithm. Overall, the experimental findings demonstrate that increasing the number of iterations improves the calibration accuracy, with 500 iterations or more producing superior visual and quantitative results.

## 5. Conclusions

Camera calibration is a fundamental and critical task in computer vision, and its accuracy significantly impacts applications such as 3D reconstruction, object recognition, augmented reality, and autonomous driving. Due to the complexity of solving nonlinear equations, traditional calibration methods can often only yield approximate solutions, leading to large reprojection errors.

To address this, a hybrid camera calibration optimization algorithm based on the star-crow optimization algorithm was proposed in this paper. By incorporating chaos mapping and sine cosine optimization strategies, the proposed NCS hybrid algorithm improves calibration accuracy and stability. Experimental results demonstrate its effectiveness in reducing reprojection error, achieving faster convergence, and offering superior global search capability compared to SSA, NOA, PSO, SMA, and ORSMA.

However, it is important to note that the enhanced accuracy comes at the cost of increased computational overhead due to the hybrid structure and iterative nature of the optimization process. In time-sensitive applications, this trade-off between accuracy and runtime must be carefully considered.

For future work, the algorithm can be extended to support multi-camera systems by incorporating inter-camera geometric constraints during the calibration process. Moreover, adaptation to dynamic environments with moving calibration targets can be explored by integrating real-time tracking and incremental calibration strategies. Additionally, optimizing parameter settings and reducing computational costs through parallel implementation could further enhance its applicability to complex and large-scale vision tasks.

## Figures and Tables

**Table 1:** Calibration results of Zhang Zhengyou calibration method.

Parameter	Zhang’s Calibration Method
$f_{x}$ (pixel)	1219.2580
$f_{y}$ (pixel)	1291.4706
$u_{0}$ (pixel)	1010.4831
$v_{0}$ (pixel)	514.4578
$k_{1}$	−0.2854
$k_{2}$	0.1147
$p_{1}$	−0.0022
$p_{2}$	−0.0027
$k_{3}$	−0.0027
Error (pixel)	18.9793

**Table 2:** Calibration results of camera internal parameters after 100 iterations.

Parameter	Algorithm					
	**SSA**	**NCS**	**NOA**	**PSO**	**SMA**	**ORSMA**
$f_{x}$ (pixel)	1215.6746	1220.5062	1220.7717	1215.7684	1209.3621	1219.7476
$f_{y}$ (pixel)	1288.2704	1292.5917	1293.4156	1290.4112	1286.5391	1289.1343
$u_{0}$ (pixel)	1005.7919	1010.5557	1010.3238	1011.9349	1009.2207	1010.6172
$v_{0}$ (pixel)	507.1347	514.2224	514.2952	513.758	515.0496	514.5245
$k_{1}$	−0.2465	−0.2916	−0.2956	−0.2917	−0.2826	−0.2846
$k_{2}$	0.055	0.1266	0.1258	0.1208	0.1156	0.1049
$p_{1}$	0.0129	−0.0018	−0.0025	−0.0017	−0.0004	−0.0011
$p_{2}$	0.002	−0.0027	−0.0024	−0.003	−0.0001	−0.0026
$k_{3}$	−0.0246	−0.0411	−0.0239	−0.0409	−0.0257	−0.0242
error	0.7586	0.0714	0.0841	0.1787	0.2584	0.0793

**Table 3:** Calibration results of camera internal parameters after 200 iterations.

Parameter	Algorithm					
	**SSA**	**NCS**	**NOA**	**PSO**	**SMA**	**ORSMA**
$f_{x}$ (pixel)	1217.6056	1219.1188	1217.2234	1220.3455	1215.6967	1220.2164
$f_{y}$ (pixel)	1290.3167	1291.3983	1289.9667	1294.2341	1283.6624	1293.1258
$u_{0}$ (pixel)	1007.3895	1010.4631	1010.3379	1010.3434	1009.9515	1010.5354
$v_{0}$ (pixel)	507.9402	514.4933	514.7585	512.9969	514.253	514.1173
$k_{1}$	−0.3519	−0.2854	−0.2745	−0.2825	−0.2755	−0.2923
$k_{2}$	0.2485	0.1149	0.1015	0.0655	0.0939	0.1222
$p_{1}$	0.0222	−0.0024	−0.0031	−0.0001	0.003	−0.0018
$p_{2}$	0.0073	−0.0027	−0.0026	−0.0029	−0.0009	−0.0027
$k_{3}$	−0.0239	−0.033	−0.0361	0.0257	−0.0252	−0.0309
error	0.8104	0.069	0.0761	0.1241	0.1665	0.0732

**Table 4:** Calibration results of camera internal parameters after 500 iterations.

Parameter	Algorithm					
	**SSA**	**NCS**	**NOA**	**PSO**	**SMA**	**ORSMA**
$f_{x}$ (pixel)	1221.7508	1219.1898	1219.3101	1221.0396	1214.1091	1219.596
$f_{y}$ (pixel)	1294.3923	1291.4735	1291.6169	1285.6581	1285.3101	1291.8986
$u_{0}$ (pixel)	1012.3229	1010.4797	1010.4771	1010.5006	1009.8975	1010.4832
$v_{0}$ (pixel)	510.3657	514.4491	514.4371	514.467	514.1085	514.4296
$k_{1}$	−0.322	−0.2851	−0.2862	−0.3006	−0.2567	−0.2884
$k_{2}$	0.1585	0.114	0.1173	0.196	0.0603	0.123
$p_{1}$	0.0019	−0.0023	−0.0023	0.0008	−0.0001	−0.0023
$p_{2}$	−0.0077	−0.0027	−0.0027	−0.0016	−0.0007	−0.0027
$k_{3}$	−0.0388	−0.0331	−0.036	−0.1282	−0.0242	−0.0402
error	0.4483	0.0687	0.0687	0.171	0.1453	0.0688

**Table 5:** Calibration results of camera internal parameters after 1000 iterations.

Parameter	Algorithm					
	**SSA**	**NCS**	**NOA**	**PSO**	**SMA**	**ORSMA**
$f_{x}$ (pixel)	1219.6206	1219.1565	1219.5507	1218.5311	1209.2597	1219.513
$f_{y}$ (pixel)	1292.452	1291.4275	1291.8728	1295.4789	1281.4706	1291.8806
$u_{0}$ (pixel)	1009.0566	1010.4806	1010.4773	1010.4614	1009.8382	1010.4817
$v_{0}$ (pixel)	508.7808	514.4473	514.431	516.0571	514.7698	514.4296
$k_{1}$	−0.3419	−0.2847	−0.2887	−0.3043	−0.2199	−0.2885
$k_{2}$	0.162	0.1123	0.1256	0.1707	0.0008	0.1249
$p_{1}$	0.0172	−0.0023	−0.0023	−0.0055	−0.0018	−0.0023
$p_{2}$	0.007	−0.0027	−0.0027	−0.0027	−0.0008	−0.0027
$k_{3}$	−0.0239	−0.0316	−0.0437	−0.0566	−0.0239	−0.0432
error	0.5854	0.0687	0.0688	0.1788	0.1869	0.0688

## Data Availability

The original contributions presented in the study are included in the article, further inquiries can be directed to the corresponding author.

## References

[1] Sun C., Ma Y., Zhang G., Gao Y., Yu Q. (2023). Calibration of Telephoto Camera Based on Affine Approximation Projection Model. Acta Opt. Sin..

[2] Zhang X., Lv T., Wang D., Zhang M. (2024). High-precision binocular camera calibration method based on a 3D calibration object. Appl. Opt..

[3] Lai X., Yang X., Zhang Q. (2023). Adaptive EKF-Based Camera Calibration Optimization Method. Acta Opt. Sin..

[4] Ma L., Chen Y.Q., Moore K.L. (2006). Analytical piecewise radial distortion model for precision camera calibration. IEEE Proc. Vis. Image Signal Process..

[5] Jetsu T., Heikkinen V., Parkkinen J., Hauta-Kasari M., Martinkauppi B., Lee S.D., Ok H.W., Kim C.Y. (2012). Color calibration of digital camera using polynomial transformation. J. Imaging Sci. Technol..

[6] Liao K., Nie L., Huang S., Lin C., Zhang J., Zhao Y., Gabbouj M., Tao D. (2023). Deep learning for camera calibration and beyond: A survey. arXiv.

[7] Li J., Yang Y., Fu G. Camera self-calibration method based on GA-PSO algorithm. Proceedings of the 2011 IEEE International Conference on Cloud Computing and Intelligence Systems.

[8] Zhang Z. Flexible Camera Calibration by Viewing a Plane from Unknown Orientations. Proceedings of the Seventh IEEE International Conference on Computer Vision.

[9] Kennedy J., Eberhart R. Particle Swarm Optimization. Proceedings of the International Conference on Neural Networks.

[10] Shi Y., Gao H., Wu D. An improved differential evolution algorithm with novel mutation strategy. Proceedings of the 2014 IEEE Symposium on Differential Evolution (SDE).

[11] Wilczewski J.M., Sahin F. A hybrid genetic scatter search algorithm using genetic screening. Proceedings of the 2009 Fifth International Conference on Soft Computing, Computing with Words and Perceptions in System Analysis, Decision and Control.

[12] Wu C., Cong M. A Multi Strategy Improved Sparrow Search Algorithm. Proceedings of the IEEE 3rd International Conference on Electronic Technology, Communication and Information.

[13] Wang C., Wang S., He X. An Improved Sine Cosine Algorithm. Proceedings of the 2023 3rd International Conference on Intelligent Communications and Computing.

[14] Qi Z.-S., Wang Z., Huang J.-H., Xue Q., Gao J.-M. (2016). Research on System Calibration of Structured-Light Measurement Based on Neural Network. Acta Photonica Sin..

[15] Du S., Wang J., Guo J. (2021). Multicamera Calibration Optimization Method Based on Improved Seagull Algorithm. Secur. Commun. Netw..

[16] Guan C., Wang X., Guan S., Ding J., He Z., Tang G. (2022). Research on Camera Calibration Optimization Method Based on Chaotic Sparrow Search Algorithm. Int. Conf. Biomed. Intell. Syst..

[17] Wei F., Wu L. (2023). Camera Calibration Based on Improved Differential Evolution Particle Swarm. Meas. Control.

[18] Sha X., Qian F., He H. (2024). Research on Improved Differential Evolution Particle Swarm Hybrid Optimization Method and Its Application in Camera Calibration. Mathematics.

[19] Abdel-Basset M., Mohamed R., Jameel M., Abouhawwash M. (2023). Nutcracker Optimizer: A Novel Nature-Inspired Metaheuristic Algorithm for Global Optimization and Engineering Design Problems. Knowl.-Based Syst..

[20] Mirjalili S. (2016). SCA: A Sine Cosine Algorithm for Solving Optimization Problems. Knowl.-Based Syst..

[21] Zhang Z. (2000). A flexible new technique for camera calibration. IEEE Trans. Pattern Anal. Mach. Intell..

[22] OpenCV Documentation: Camera Calibration. https://docs.opencv.org.

